# Multi-Label Feature Selection Combining Three Types of Conditional Relevance

**DOI:** 10.3390/e23121617

**Published:** 2021-12-01

**Authors:** Lingbo Gao, Yiqiang Wang, Yonghao Li, Ping Zhang, Liang Hu

**Affiliations:** 1 College of Computer Science and Technology, Jilin University, Changchun 130012, China; gaolb19@mails.jlu.edu.cn (L.G.); yiqiang19@mails.jlu.edu.cn (Y.W.); yonghao17@mails.jlu.edu.cn (Y.L.); zhangping18@mails.jlu.edu.cn (P.Z.); 2 Key Laboratory of Symbolic Computation and Knowledge Engineering of Ministry of Education, Jilin University, Changchun 130012, China

**Keywords:** feature selection, information theory, feature relevance, label-related feature redundancy, conditional relevance

## Abstract

With the rapid growth of the Internet, the curse of dimensionality caused by massive multi-label data has attracted extensive attention. Feature selection plays an indispensable role in dimensionality reduction processing. Many researchers have focused on this subject based on information theory. Here, to evaluate feature relevance, a novel feature relevance term (FR) that employs three incremental information terms to comprehensively consider three key aspects (candidate features, selected features, and label correlations) is designed. A thorough examination of the three key aspects of FR outlined above is more favorable to capturing the optimal features. Moreover, we employ label-related feature redundancy as the label-related feature redundancy term (LR) to reduce unnecessary redundancy. Therefore, a designed multi-label feature selection method that integrates FR with LR is proposed, namely, Feature Selection combining three types of Conditional Relevance (TCRFS). Numerous experiments indicate that TCRFS outperforms the other 6 state-of-the-art multi-label approaches on 13 multi-label benchmark data sets from 4 domains.

## 1. Introduction

In recent years, multi-label learning [1,2,3,4] has been increasingly popular in applications such as text categorization [5], image annotation [6], protein function prediction [7], etc. Additionally, feature selection is of great significance to solving industrial application problems. Some researchers monitor the wind speed in the wake region to detect wind farm faults based on feature selection [8]. In signal processing applications, feature selection is effective for chatter vibration diagnosis in CNC machines [9]. Feature selection is adopted to classify the cutting stabilities based on the selected features [10]. The most crucial thing in diverse multi-label applications is to classify each sample and its corresponding label accurately. Multi-label learning, such as traditional classification approaches, is vulnerable to dimensional catastrophes. The number of features in text multi-label data is frequently in the tens of thousands, which means that there are a lot of redundant or irrelevant features [11,12]. It can easily lead to the “curse of dimensionality”, which dramatically increases the model complexity and computation time [13]. Feature selection is the process of selecting a set of feature subsets with distinguishing features from the original data set according to specific evaluation criteria. Redundant or irrelevant features can be eliminated to improve model accuracy and reduce feature dimensions, feature space, and running time [14,15]. Simultaneously, the selected features are more conducive to model understanding and data analysis.

In traditional machine learning problems, feature selection approaches include wrapper, embedded, and filter approaches [16,17,18,19]. Among them, wrapper feature selection approaches use the classifier performance to weigh the pros and cons of a feature subset, which has high computational complexity and a large memory footprint [20,21]. The processes of feature selection and learner training are combined in embedded approaches [22,23]. Feature selection is automatically conducted during the learner training procedure when the two are completed in the same optimization procedure. Filter feature selection approaches weigh the pros and cons of feature subsets using specific evaluation criteria [24,25]. It is independent of the classifier, and the calculation is fast and straight. As a result, the filter feature selection approaches are generally used for feature selection.

There are also the above-mentioned three feature selection approaches in multi-label feature selection, with filter feature selection being the most popular. Information theory is a standard mathematical tool for filter feature selection [26]. Based on information theory, this paper mainly focuses on three key aspects that affect feature relevance: candidate features, selected features, and label correlations. The method proposed in this paper examines the amount of information shared between the selected feature subset and the total label set to evaluate feature relevance and denotes it as ΔI for the time being. Once any candidate feature is selected in the current selected feature subset, the current selected feature subset will be updated at this point, and ΔI will be altered accordingly. Moreover, the original label correlations in the total label set also affect ΔI due to some new candidate features being added to the current selected feature subset. Hence, three incremental information terms which combine candidate features, selected features, and label correlations to evaluate feature relevance are used to design a novel feature relevance term. Furthermore, we employ label-related feature redundancy as the feature redundancy term to reduce unnecessary redundancy. Table 1 provides three abbreviations and their corresponding meanings we mentioned. We explain them in detail in Section 4.

The major contributions of this paper are as follows:

1. Analyze and discuss the indispensability of the three key aspects (candidate features, selected features and label correlations) for feature relevance evaluation;
2. Three incremental information terms taking three key aspects into account are used to express three types of conditional relevance. Then, FR combining the three incremental information terms is designed;
3. A designed multi-label feature selection method that integrates FR with LR is proposed, namely TCRFS;
4. TCRFS is compared to 6 state-of-the-art multi-label feature selection methods on 13 benchmark multi-label data sets using 4 evaluation criteria and certified the efficacy in numerous experiments.

The rest of this paper is structured as follows. Section 2 introduces the preliminary theoretical knowledge of this paper: information theory and the four evaluation criteria used in our experiments. Related works are reviewed in Section 3. Section 4 combines three types of conditional relevance to design FR and proposes TCRFS, which integrates FR with LR. The efficacy of TCRFS is proven by comparing it with 6 multi-label methods on 13 benchmark data sets applying 4 evaluation criteria in Section 5. Section 6 concludes our work in this paper.

## 2. Preliminaries

### 2.1. Information Theory for Multi-Label Feature Selection

Information theory is a popular and effective means to tackle the problem of multi-label feature selection [27,28,29]. It is used to measure the correlation between random variables [30] and its fundamentals are covered in this subsection.

Assume that the selected feature subset $S=\{f_{1},f_{2},\ldots ,f_{n}\}$, the label set $L=\{l_{1},l_{2},\ldots ,l_{m}\}$. To convey feature relevance, we typically employ I(S;L), which is mutual information between the selected feature subset and the total label set. Mutual information is a measure in information theory. It can be seen as the amount of information contained in one random variable about another random variable. Assume two discrete random variables $X=\{x_{1},x_{2},\ldots ,x_{n}\}$, $Y=\{y_{1},y_{2},\ldots ,y_{m}\}$, then the mutual information between *X* and *Y* can be represented as I(X;Y). Its expansion formula is as follows:

(1) $$\begin{array}{c} I(X;Y)=H(X)-H(X|Y)=H(Y)-H(Y|X) \end{array}$$

where H(X) denotes the information entropy of *X*, and H(X|Y) denotes the conditional entropy of *X* given *Y*. Information entropy is a concept used to measure the amount of information in information theory. H(X) is defined as:

(2) $$\begin{array}{c} H\left(X\right)=-\sum_{i=1}^{n}p\left(x_{i}\right)\log p\left(x_{i}\right) \end{array}$$

where $p(x_{i})$ represents the probability distribution of $x_{i}$, and the base of the logarithm is 2. The conditional entropy H(X|Y) is defined as the mathematical expectation of *Y* for the entropy of the conditional probability distribution of *X* under the given condition *Y*:

(3) $$\begin{array}{c} H\left(X|Y\right)=-\sum_{i=1}^{n}\sum_{j=1}^{m}p\left(x_{i},y_{j}\right)\log p\left(x_{i}|y_{j}\right) \end{array}$$

where $p(x_{i},y_{i})$ and $p(x_{i}|y_{i})$ represent the joint probability distribution of $\left(x_{i},y_{i}\right)$ and the conditional probability distribution of $x_{i}$ given $y_{i}$, respectively. H(X|Y) can also be represented as follows:

(4) $$\begin{array}{c} H(X|Y)=H(X,Y)-H(Y) \end{array}$$

where H(X,Y) is another measure in information theory, namely, the joint entropy. Its definition is as follows:

(5) $$\begin{array}{c} H\left(X,Y\right)=-\sum_{i=1}^{n}\sum_{j=1}^{m}p\left(x_{i},y_{j}\right)\log p\left(x_{i},y_{j}\right) \end{array}$$

According to Equation (4), combining the relationship between the three different measures of the amount information, the mutual information I(X;Y) can also be alternatively written as follows:

(6) $$\begin{array}{c} I(X;Y)=H(X)+H(Y)-H(X,Y) \end{array}$$

It is common in multi-label feature selection to have more than two random variables, assuming another discrete random variable $Z=\{z_{1},z_{2},\ldots ,z_{q}\}$. The conditional mutual information I(X;Y|Z), which expresses the expected value of mutual information of two discrete random variables *X* and *Y* given the value of the third discrete variable *Z*. It is represented as follows:

(7) $$\begin{array}{c} I(X;Y|Z)=I(X,Z;Y)-I(Y;Z)=I(X;Y,Z)-I(X;Z)=I(X;Y)-I(X;Y;Z) \end{array}$$

where I(X,Z;Y) is the joint mutual information and I(X;Y;Z) is the interaction information. Their expansion formulas are as follows:

(8) I(X,Z;Y)=I(X;Y|Z)+I(Y;Z)=I(Y;Z|X)+I(X;Y)

(9) I(X;Y;Z)=I(X;Y)+I(X;Z)−I(X;Y,Z)=I(X;Y)−I(X;Y|Z)

### 2.2. Evaluation Criteria for Multi-Label Feature Selection

In our experiments, we employ four distinct evaluation criteria to confirm the efficacy of TCRFS. The four evaluation criteria are essentially separated into two categories: label-based evaluation criteria and example-based evaluation criteria [31]. The label-based evaluation criteria include Macro-$F_{1}$ and Micro-$F_{1}$ [32]. The higher the value of these two indicators, the better the classification effect. Macro-$F_{1}$ actually calculates the $F_{1}$-score of *q* categories first and then averages it as follows:

(10) $$\begin{array}{c} \mathrm{Macro}\text{-}F_{1}=\frac{1}{q}\sum_{i=1}^{q}\frac{2TP_{i}}{2TP_{i}+FP_{i}+FN_{i}} \end{array}$$

where $TP_{i}$, $FP_{i}$, and $FN_{i}$ represent true positives, false positives, and false negatives in *i*-th category, respectively. Micro-$F_{1}$ calculates the confusion matrix of each category, and adds the confusion matrix to obtain a multi-category confusion matrix and then calculates the $F_{1}$-score as follows:

(11) $$\begin{array}{c} \mathrm{Macro}\text{-}F_{1}=\frac{\sum_{i=1}^{q}2TP_{i}}{\sum_{i=1}^{q}\left(2TP_{i}+FP_{i}+FN_{i}\right)} \end{array}$$

The example-based evaluation criteria include the Hamming Loss (HL) and Zero One Loss (ZOL) [33]. The lower the value of these two indicators, the better the classification effect. HL is a metric for the number of times a label is misclassified. That is, a label belonging to a sample is not predicted, and a label not belonging to the sample is projected to belong to the sample. Suppose that $D=\{\left(x_{i},Y_{i}\right)|1\leq i\leq m\}$ is a label test set and $Y_{i}\subseteq Y$ is a set of class labels corresponding to $x_{i}$, where Y is the label space with *q* categories. The definition of HL is as follows:

(12) $$\begin{array}{c} HL=\frac{1}{m}\sum_{i=1}^{m}\frac{Y_{i}^{{}^{\prime }}⊕Y_{i}}{q} \end{array}$$

where ⊕ means the XOR operation. $Y_{i}^{{}^{\prime }}$ denotes the predicted label set corresponding to $x_{i}$. The other example-based criterion ZOL is defined as follows:

(13) $$\begin{array}{c} ZOL=\frac{1}{m}\sum_{i=1}^{m}\delta \left(argmax_{y\in Y}h\left(x_{i},y\right)\right) \end{array}$$

If the predicted label subset and the true label subset match, the ZOL score is 1 (i.e., δ=1), but if there is no error, the score is 0 (i.e., δ=0).

## 3. Related Work

There have been a lot of multi-label learning algorithms proposed so far. These multi-label learning algorithms can be divided into problem transform and algorithm adaptation [34,35]. Problem transform is the conversion of multi-label learning into traditional single-label learning, such as Binary Relevance (BR) [36], Pruned Problem Transformation (PPT) [37], and Label Power (LP) [38]. BR treats the prediction of each label as an independent single classification issue and trains an individual classifier for each label with all of the training data [33]. However, it ignores the relationships between the labels, so it is possible to end up with imbalanced data. PPT removes the labels with a low frequency by considering the label set with a predetermined minimum number of occurrences. However, this irreversible conversion will result in the loss of class information [39].

In contrast to problem transform, algorithm adaptation directly enhances the existing single-label data learning algorithms to adapt to multi-label data processing. Algorithm adaption improves the issues caused by problem transformation. Cai et al. [40] propose Robust and Pragmatic Multi-class Feature Selection (RALM-FS) based on an augmented Lagrangian method, where there is just one $\ell_{2,1}$-norm loss term in RALM-FS, with an apparent $\ell_{2,0}$-norm equality constraint. Lee and Kim [41] propose the D2F method that makes use of interactive information based on mutual information. It is capable of measuring multiple variable dependencies by default, and its definition is as follows:

(14) $$\begin{array}{c} J\left(f_{k}\right)=\sum_{l_{i}\in L}I\left(f_{k};l_{i}\right)-\sum_{f_{j}\in S}\sum_{l_{i}\in L}I\left(f_{k};f_{j};l_{i}\right) \end{array}$$

where $\sum_{l_{i}\in L}I\left(f_{k};l_{i}\right)$ and $\sum_{f_{j}\in S}\sum_{l_{i}\in L}I\left(f_{k};f_{j};l_{i}\right)$ are regarded as the feature relevance term and the feature redundancy term, respectively. The feature relevance of D2F only considers the candidate features in feature relevance, which ignores selected features and label correlations. Lee and Kim [42] propose the Pairwise Multi-label Utility (PMU), which is derived from I(S;L) as follows:

(15) $$\begin{array}{c} J\left(f_{k}\right)=\sum_{l_{i}\in L}I\left(f_{k};l_{i}\right)-\sum_{f_{j}\in S}\sum_{l_{i}\in L}I\left(f_{k};f_{j};l_{i}\right)-\sum_{l_{i}\in L}\sum_{l_{j}\in L}I\left(f_{k};l_{i};l_{j}\right) \end{array}$$

where $\sum_{l_{i}\in L}I\left(f_{k};l_{i}\right)$ is to measure the feature relevance and $\sum_{f_{j}\in S}\sum_{l_{i}\in L}I\left(f_{k};f_{j};l_{i}\right)+$$\sum_{l_{i}\in L}\sum_{l_{j}\in L}I\left(f_{k};l_{i};l_{j}\right)$ is to measure the feature redundancy. Afterward, Lee and Kim [43] propose multi-label feature selection based on a scalable criterion for large SCLS. SCLS uses a scalable relevance evaluation approach to assess conditional relevance more correctly:

(16) $$\begin{array}{c} J\left(f_{k}\right)=\sum_{l_{i}\in L}I\left(f_{k};l_{i}\right)-\sum_{f_{j}\in S}\frac{I(f_{k};f_{j})}{H(f_{k})}\sum_{l_{i}\in L}I\left(f_{k};l_{i}\right)=\left[1-\sum_{f_{j}\in S}\frac{I(f_{k};f_{j})}{H(f_{k})}\right]\sum_{l_{i}\in L}I\left(f_{k};l_{i}\right) \end{array}$$

In fact, the scalable relevance in SCLS considers both candidate features and selected features but ignores label correlations. Liu et al. [44] propose feature selection for multi-label learning with streaming label (FSSL) in which label-specific features are learned for each newly received label, and then label-specific features are fused for all currently received labels. Lin et al. [45] apply a multi-label feature selection method based on fuzzy mutual information (MUCO) to the redundancy and correlation analysis strategies. The next feature that enters *S* can be selected by the following:

(17) $$\begin{array}{c} J\left(f_{k}\right)=FMI\left(f_{k};L\right)-\frac{1}{\left|S\right|}\sum_{f_{j}\in S}\left(FMI\left(f_{k};f_{j}\right)\right) \end{array}$$

where $FMI(f_{k};L)$ denotes the fuzzy mutual information.

When we try to add a new candidate feature $f_{k}$ to the current selected feature subset *S*, the feature $f_{k}$, the selected features $f_{j}$ in *S*, and label correlations in the total label set will all impact feature relevance. To this end, FR is devised by merging the three types of conditional relevance. Therefore, a designed multi-label feature selection method TCRFS that integrates FR with LR is proposed.

## 4. TCRFS: Feature Selection Combining Three Types of Conditional Relevance

According to the past multi-label feature selection methods, they do not take into account all the three key aspects of influencing feature relevance. That is, the key aspects that influence feature relevance are not comprehensively examined. Here, we utilize three incremental information terms to depict three types of conditional relevance that consider candidate features, selected features, and label correlations comprehensively. The reasons for our consideration are as follows.

### 4.1. The Three Key Aspects of Feature Relevance We Consider

#### 4.1.1. Candidate Features

**Figure 1:**
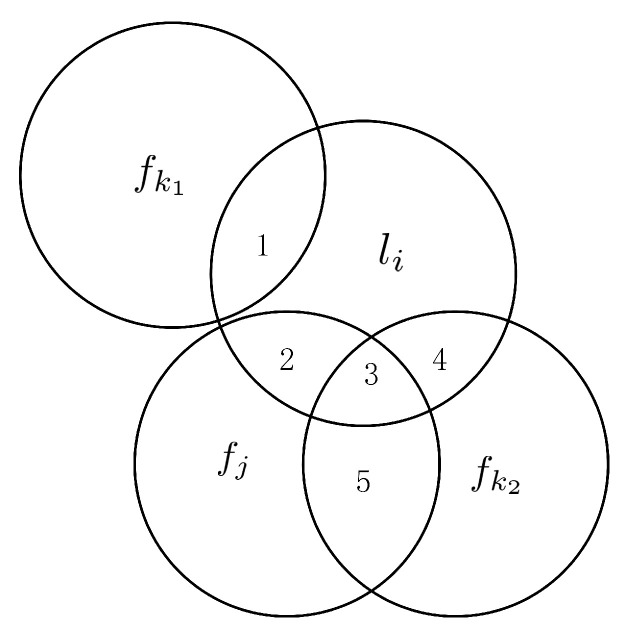
The relationship between features and labels in the Venn diagram.

We evaluate each candidate feature according to specific criteria. When a candidate feature $f_{k}$ attempts to enter the current selected feature subset *S* as a new selected feature to generate a new selected feature subset, it will affect the amount of information provided by the current selected feature subset to the label set. The influence of candidate features is represented by a Venn diagram, as shown in Figure 1.

In Figure 1, we assume that $f_{k_{1}}$ and $f_{k_{2}}$ are two candidate features, $f_{j}$ is a selected feature in *S*, and $l_{i}$ is a label in the total label set *L*. $f_{k_{1}}$ is irrelevant to $f_{j}$, and $f_{k_{2}}$ is redundant with $f_{j}$. The amount of information provided by $f_{j}$ to $l_{i}$ is mutual information $I(f_{j};l_{i})$, that is, the area {2,3}. If $f_{k_{1}}$ is selected, then the amount of information provided by $f_{j}$ to $l_{i}$ will be $I(f_{j};l_{i}|f_{k_{1}})$, which corresponds to the area {2,3}. If $f_{k_{2}}$ is selected, then the amount of information provided by $f_{j}$ to $l_{i}$ will be $I(f_{j};l_{i}|f_{k_{2}})$, which corresponds to the area {2} since the area {2} is less than the area {2,3}, $I\left(f_{j};l_{i}|f_{k_{2}}\right)<I\left(f_{j};l_{i}|f_{k_{1}}\right)$. Therefore, the higher the label-related redundancy between the candidate feature and the selected feature in the current selected feature subset, the greater the amount of information between $f_{j}$ and $l_{i}$ is reduced. In other words, the label-related redundancy between the candidate feature and the selected features should be kept to a minimum. From this point of view, $f_{k_{1}}$ takes precedence over $f_{k_{2}}$.

#### 4.1.2. Selected Features

**Figure 2:**
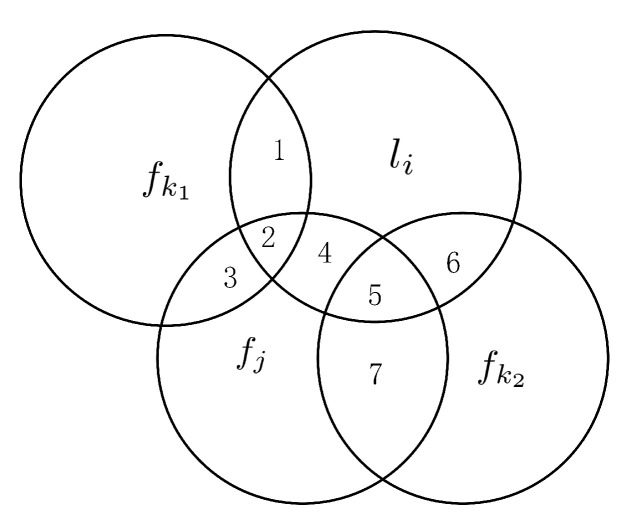
The relationship between features and labels in the Venn diagram.

The influence of selected features is represented by a Venn diagram as shown in Figure 2.

As shown in Figure 2, $f_{k_{1}}$ and $f_{k_{2}}$ are both redundant with $f_{j}$. Without considering selected features, the information that $f_{k_{1}}$ and $f_{k_{2}}$ shared with the label $l_{i}$ are $I(f_{k_{1}};l_{i})$ and $I(f_{k_{2}};l_{i})$, respectively. The area {1,2} denotes $I(f_{k_{1}};l_{i})$, and the area {5,6} denotes $I(f_{k_{2}};l_{i})$. We assume that the area {1,2} is less than the area {5,6}, the area {2} is less than the area {5}, but the area {1} is larger than {6}. With the selected features taken into account, the information shared by $f_{k_{1}}$ and $l_{i}$ is $I(f_{k_{1}};l_{i}|f_{j})$ (i.e., the area {1}), and the information shared by $f_{k_{2}}$ and $l_{i}$ is $I(f_{k_{2}};l_{i}|f_{j})$ (i.e., the area {6}): $I\left(f_{k_{1}};l_{i}\right)<I\left(f_{k_{2}};l_{i}\right)$, but $I\left(f_{k_{1}};l_{i}|f_{j}\right)>I\left(f_{k_{2}};l_{i}|f_{j}\right)$. There are two causes for this situation, the first is that the amount of information provided to $l_{i}$ by $f_{k_{2}}$ itself is insufficient, and the second is that the label-related redundancy between $f_{k_{2}}$ and $f_{j}$ is excessive. Now, in the hypothesis, replace the condition that area {1} is larger than the area {6} to the area {1} is less than the area {6}, and we obtain the following result: $I\left(f_{k_{1}};l_{i}\right)<I\left(f_{k_{2}};l_{i}\right)$ but $I\left(f_{k_{1}};l_{i}|f_{j}\right)<I\left(f_{k_{2}};l_{i}|f_{j}\right)$. Therefore, considering the influence of the selected features on feature relevance is necessary.

#### 4.1.3. Label Correlations

**Figure 3:**
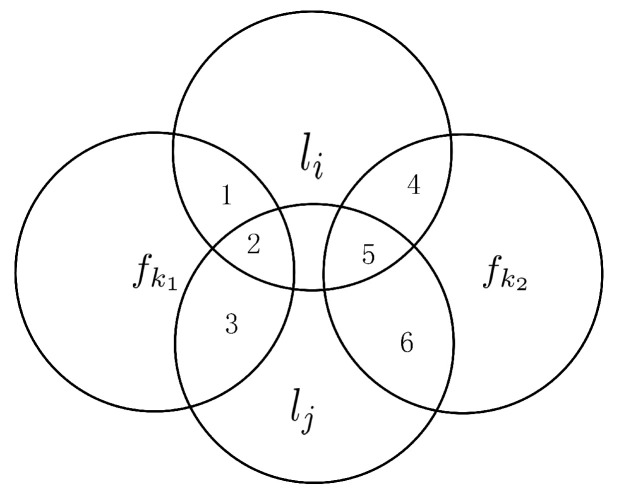
The relationship between features and labels in the Venn diagram.

It has no influence on the amount of information between candidate features and each label if the labels are independent. The influence of label correlations is represented by a Venn diagram as shown in Figure 3.

In Figure 3, $l_{i}$ and $l_{j}$ are two redundant labels, that is, there exists a correlation between $l_{i}$ and $l_{j}$. Without the consideration of label correlations, the amount of information provided to $l_{i}$ by $f_{k_{1}}$ is $I(f_{k_{1}};l_{i})$ (the area {1,2}) and the amount of information provided to $l_{i}$ by $f_{k_{2}}$ is $I(f_{k_{2}};l_{i})$ (the area {4,5}). Then, while taking label correlations into consideration, the amount of information provided to $l_{i}$ by $f_{k_{1}}$ is $I(f_{k_{1}};l_{i}|l_{j})$ (the area {1}) and the amount of information provided to $l_{i}$ by $f_{k_{2}}$ is $I(f_{k_{2}};l_{i}|l_{j})$ (the area {4}). Now, provide the first hypothesis: the area {1,2} is larger than the area {4,5}, the area {2} is larger than the area {5}, but the area {1} is less than the area {4}. Hence, $I\left(f_{k_{1}};l_{i}\right)>I\left(f_{k_{2}};l_{i}\right)$ but $I\left(f_{k_{1}};l_{i}|l_{j}\right)<I\left(f_{k_{2}};l_{i}|l_{j}\right)$. The second hypothesis modifies the last condition in the first hypothesis: the area {1} is larger than the area {4}. Hence, $I\left(f_{k_{1}};l_{i}\right)>I\left(f_{k_{2}};l_{i}\right)$ and $I\left(f_{k_{1}};l_{i}|l_{j}\right)>I\left(f_{k_{2}};l_{i}|l_{j}\right)$. We call the area {2} and the area {5} feature-related label redundancy. Therefore, the original amount of information between candidate features and labels and the feature-related label redundancy can affect the selection of features. Merely using the accumulation of mutual information as the feature relevance will cause the redundant recalculation of feature-related label redundancy.

According to the three key aspects of feature relevance described above, they are indispensable. As a result, we devise FR as the feature relevance term of TCRFS.

### 4.2. Evaluation Function of TCRFS

#### 4.2.1. Definitions of FR and LR

Regarding the feature relevance evaluation, we distinguish the importance of features based on the closeness of the relationship between features and labels. According to Section 4.1, candidate features, selected features, and label correlations are three key aspects on evaluating feature relevance. In order to be able to perform better in multi-label classification, we utilize three types of conditional relevance ($I(f_{k};l_{i}|f_{j})$, $I(f_{j};l_{i}|f_{k})$ and $I(f_{k};l_{i}|l_{j}$) to represent the feature relevance term in the proposed method. By using three incremental information terms to summarize the three key aspects of feature relevance, FR is devised. The three incremental information terms represent the three respective types of conditional relevance.

**Definition 1.**  *(FR). Suppose that $F=\{f_{1},f_{2},\ldots ,f_{m}\}$ and $L=\{l_{1},l_{2},\ldots ,1_{n}\}$ are the total feature set and the total label set, respectively. Let S be the selected feature set excluding candidate features, that is, $f_{k}\in F-S$. FR is depicted as follows:*

(18) $$\begin{array}{c} FR\left(f_{k}\right)=\sum_{l_{i}\in L}\sum_{f_{j}\in S}I\left(f_{k};l_{i}|f_{j}\right)+\sum_{l_{i}\in L}\sum_{f_{j}\in S}I\left(f_{j};l_{i}|f_{k}\right)+\sum_{l_{i}\in L}\sum_{i\neq j,l_{j}\in L}I\left(f_{k};l_{i}|l_{j}\right) \end{array}$$

*where $\sum_{l_{i}\in L}\sum_{f_{j}\in S}I\left(f_{k};l_{i}|f_{j}\right)$ denotes the conditional relevance taking candidate features into account while evaluating feature relevance, $\sum_{l_{i}\in L}\sum_{f_{j}\in S}I\left(f_{j};l_{i}|f_{k}\right)$ denotes the conditional relevance taking selected features into account while evaluating feature relevance, and $\sum_{l_{i}\in L}\sum_{i\neq j,l_{j}\in L}I\left(f_{k};l_{i}|l_{j}\right)$ denotes the conditional relevance taking label correlations into account while evaluating feature relevance. The comprehensive evaluation of the above-mentioned three key aspects of feature relevance is more conducive to capturing the optimal features. Furthermore, FR can be expanded as follows:*

(19) $$\begin{array}{cc} FR(f_{k})= & \sum_{l_{i}\in L}\sum_{f_{j}\in S}I\left(f_{k};l_{i}|f_{j}\right)+\sum_{l_{i}\in L}\sum_{f_{j}\in S}I\left(f_{j};l_{i}|f_{k}\right)+\sum_{l_{i}\in L}\sum_{i\neq j,l_{j}\in L}I\left(f_{k};l_{i}|l_{j}\right)\\ = & \sum_{l_{i}\in L}\sum_{f_{j}\in S}\left[I\left(f_{k};l_{i}|f_{j}\right)+I\left(f_{j};l_{i}|f_{k}\right)\right]+\sum_{l_{i}\in L}\sum_{i\neq j,l_{j}\in L}I\left(f_{k};l_{i}|l_{j}\right)\\ = & \sum_{l_{i}\in L}\sum_{f_{j}\in S}\left[I\left(f_{k},f_{j};l_{i}\right)-I\left(f_{j};l_{i}\right)+I\left(f_{j},f_{k};l_{i}\right)-I\left(f_{k};l_{i}\right)\right]+\sum_{l_{i}\in L}\sum_{i\neq j,l_{j}\in L}\left[I\left(f_{k};l_{i},l_{j}\right)-I\left(l_{i};l_{j}\right)\right]\\ \propto & \sum_{l_{i}\in L}\sum_{f_{j}\in S}\left[2I\left(f_{k},f_{j};l_{i}\right)-I\left(f_{k};l_{i}\right)\right]+\sum_{l_{i}\in L}\sum_{i\neq j,l_{j}\in L}I\left(f_{k};l_{i},l_{j}\right) \end{array}$$

*where $I(f_{j};l_{i})$ and $I(l_{i};l_{j})$ are considered to be two constants in feature selection.*

**Definition 2.**  *(LR). In the initial analysis of the three key aspects of feature relevance, it is mentioned that the label-related feature redundancy is repeatedly calculated in the previous methods, which will impact on capturing the optimal features. Here, LR is devised as follows:*

(20) $$\begin{array}{c} LR\left(f_{k}\right)=\sum_{l_{i}\in L}\sum_{f_{j}\in S}\left[I\left(f_{k};f_{j}\right)-I\left(f_{k};f_{j}|l_{i}\right)\right] \end{array}$$

As indicated in Table 2, we have compiled a list of feature relevance terms and feature redundancy terms for TCRFS and the contrasted methods based on information theory.

#### 4.2.2. Proposed Method

We design FR and LR to analyze and discuss feature relevance and feature redundancy, respectively, in Section 4.2.1. Subsequently, TCRFS, a designed multi-label feature selection method that integrates FR with LR, is suggested. The definition of TCRFS is as follows:

(21) $$\begin{array}{cc} J(f_{k})= & \frac{1}{\left|L||S\right|}\sum_{l_{i}\in L}\sum_{f_{j}\in S}I\left(f_{k};l_{i}|f_{j}\right)+\frac{1}{\left|L||S\right|}\sum_{l_{i}\in L}\sum_{f_{j}\in S}I\left(f_{j};l_{i}|f_{j}\right)+\frac{1}{\left|L||L-1\right|}\sum_{l_{i}\in L}\sum_{i\neq j,l_{j}\in L}I\left(f_{k};l_{i}|l_{j}\right)\\ - & \frac{1}{\left|L||S\right|}\sum_{l_{i}\in L}\sum_{f_{j}\in S}\left[I\left(f_{k};f_{j}\right)-I\left(f_{k};f_{j}|l_{i}\right)\right], \end{array}$$

where |L| and |S| represent the number of the total label set and the number of the selected subset, respectively, and their inversions are $\frac{1}{\left|L\right|}$ and $\frac{1}{\left|S\right|}$, respectively. The feature relevance term and the feature redundancy term can be balanced using the two balance parameters $\frac{1}{\left|L||S\right|}$ and $\frac{1}{\left|L||L-1\right|}$. According to Formula (19), Formula (21) can be rewritten as follows:

(22) $$\begin{array}{cc} J(f_{k})= & \frac{1}{\left|L||S\right|}\sum_{l_{i}\in L}\sum_{f_{j}\in S}I\left(f_{k};l_{i}|f_{j}\right)+\frac{1}{\left|L||S\right|}\sum_{l_{i}\in L}\sum_{f_{j}\in S}I\left(f_{j};l_{i}|f_{j}\right)+\frac{1}{\left|L||L-1\right|}\sum_{l_{i}\in L}\sum_{i\neq j,l_{j}\in L}(I\left(f_{k};l_{i}|l_{j}\right)\\ \; & -\frac{1}{\left|L||S\right|}\sum_{l_{i}\in L}\sum_{f_{j}\in S}\left\{I\left(f_{k};f_{j}\right)-I\left(f_{k};f_{j}|l_{i}\right)\right\}\\ = & \frac{1}{\left|L||S\right|}\sum_{l_{i}\in L}\sum_{f_{j}\in S}\left\{I\left(f_{k};l_{i}|f_{j}\right)+I\left(f_{j};l_{i}|f_{j}\right)-I\left(f_{k};f_{j}\right)+I\left(f_{k};f_{j}|l_{i}\right)\right\}+\frac{1}{\left|L||L-1\right|}\sum_{l_{i}\in L}\sum_{i\neq j,l_{j}\in L}I\left(f_{k};l_{i}|l_{j}\right)\\ \propto & \frac{1}{\left|L||S\right|}\sum_{l_{i}\in L}\sum_{f_{j}\in S}\left\{2I\left(f_{k},f_{j};l_{i}\right)-I\left(f_{k};l_{i}\right)-I\left(f_{k};f_{j};l_{i}\right)\right\}+\frac{1}{\left|L||L-1\right|}\sum_{l_{i}\in L}\sum_{i\neq j,l_{j}\in L}I\left(f_{k};l_{i},l_{j}\right)\\ = & \frac{1}{\left|L||S\right|}\sum_{l_{i}\in L}\sum_{f_{j}\in S}\left\{2I\left(f_{k},f_{j};l_{i}\right)-I\left(f_{k};l_{i}|f_{j}\right)-2I\left(f_{k};f_{j};l_{i}\right)\right\}+\frac{1}{\left|L||L-1\right|}\sum_{l_{i}\in L}\sum_{i\neq j,l_{j}\in L}I\left(f_{k};l_{i},l_{j}\right)\\ = & \frac{1}{\left|L||S\right|}\sum_{l_{i}\in L}\sum_{f_{j}\in S}\left\{2I\left(f_{k},f_{j};l_{i}\right)-I\left(f_{k},f_{j};l_{i}\right)+I\left(f_{j};l_{i}\right)-2I\left(f_{k};f_{j};l_{i}\right)\right\}+\frac{1}{\left|L||L-1\right|}\sum_{l_{i}\in L}\sum_{i\neq j,l_{j}\in L}I\left(f_{k};l_{i},l_{j}\right)\\ \propto & \frac{1}{\left|L||S\right|}\sum_{l_{i}\in L}\sum_{f_{j}\in S}\left\{I\left(f_{k},f_{j};l_{i}\right)-2I\left(f_{k};f_{j};l_{i}\right)\right\}+\frac{1}{\left|L||L-1\right|}\sum_{l_{i}\in L}\sum_{i\neq j,l_{j}\in L}I\left(f_{k};l_{i},l_{j}\right)\\ = & \frac{1}{\left|L||S\right|}\sum_{l_{i}\in L}\sum_{f_{j}\in S}I\left(f_{k},f_{j};l_{i}\right)+\frac{1}{\left|L||L-1\right|}\sum_{l_{i}\in L}\sum_{i\neq j,l_{j}\in L}I\left(f_{k};l_{i},l_{j}\right)-\frac{2}{\left|L||S\right|}\sum_{l_{i}\in L}\sum_{f_{j}\in S}I\left(f_{k};f_{j};l_{i}\right), \end{array}$$

where $\frac{1}{\left|L||S\right|}\sum_{l_{i}\in L}\sum_{f_{j}\in S}I\left(f_{k},f_{j};l_{i}\right)+\frac{1}{\left|L||L-1\right|}\sum_{l_{i}\in L}\sum_{i\neq j,l_{j}\in L}I\left(f_{k};l_{i},l_{j}\right)$ is regarded as the new feature relevance term and $\frac{2}{\left|L||S\right|}\sum_{l_{i}\in L}\sum_{f_{j}\in S}I\left(f_{k};f_{j};l_{i}\right)$ is regarded as the new feature redundancy term. The pseudo-code of TCRFS (Algorithm 1) is as follows:
**Algorithm 1.** TCRFS.**Input:**      A training sample *D* with a full feature set $F=\{f_{1},f_{2},\ldots ,f_{n}\}$ and the label set $L=\{l_{1},l_{2},\ldots ,l_{m}\}$; User-specified threshold *K*.**Output:**      The selected feature subset *S*.
1:S←⌀;2:k←0;3:**for**i=1 to *n* **do**4:   Calculate the feature relevance $I(f_{i};l_{i}|l_{j})$;5:**end for**6:**while**k<K**do**7:   **if** *k* == 0 **then**8:       Select the first feature $f_{j}$ with the largest $I(f_{i};l_{i}|l_{j})$;9:       k=k+1;10:     $S=S\cup \{f_{j}\}$;11:     $F=F-\{f_{j}\}$;12:   **end if**13:   **for** each candidate feature $f_{i}\in F$ **do**14:     According to the Formula (21) and calculate the $J(f_{i})$;15:   **end for**16:   Select the feature $f_{j}$ with the largest $J(f_{i})$;17:   $S=S\cup \{f_{j}\}$;18:   $F=F-\{f_{j}\}$;19:   k=k+1;20:**end while**

First, in lines 1–5, the selected feature subset *S* and the number of selected features *k* in the proposed method are initialized. To capture the initial feature, we calculate the incremental information $I(f_{i};l_{i}|l_{j})$ to capture the first feature (lines 6–12). Then, until the procedure is complete, calculate and capture the following feature (lines 13–20).

### 4.3. Time Complexity

Time complexity is also one of the criteria for evaluating the pros and cons of methods. The time complexity of each contrasted method and TCRFS has been computed here. Assume that there are *n*, *p*, and *q* instances, features, and labels, respectively. The computational complexity of mutual information and conditional mutual information is O(n) for all instances that have to be visited for probability. Each iteration of RALM-FS requires $O(p^{3})$. Assume that *k* denotes the number of selected features. The time complexity of TCRFS is $O(npq^{2}+knpq)$ as three incremental information terms and one label-related feature redundancy term are calculated. Similarly, D2F, PMU, and SCLS have time complexities of O(npq+knpq), $O(npq+knpq+npq^{2})$, and O(nma+knm), respectively. FSSL has a time complexity of O(knpq). The time complexity of MUCO is $O(n^{2}+p\left(p-k\right))$ since it constructs a fuzzy matrix and incremental search.

## 5. Experimental Evaluation

**Figure 4:**
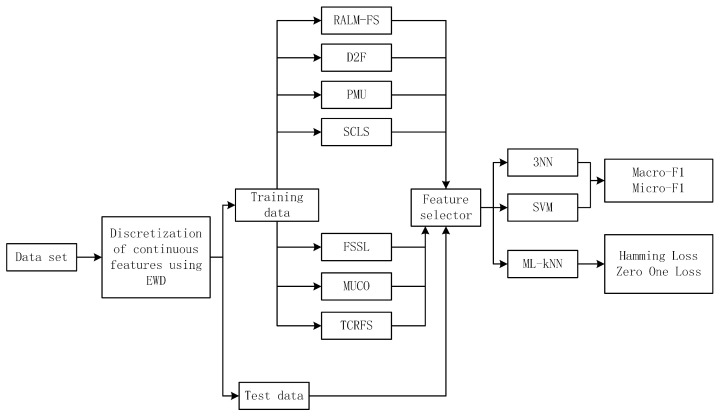
The experimental framework.

Against the demonstrated efficacy of TCRFS, we compare it to 6 advanced multi-label feature selection approaches (RALM-FS [40], D2F [41], PMU [42], SCLS [43], FSSL [44], and MUCO [45]), on 13 benchmark data sets in this section. As a result, we have conducted numerous experiments based on four different criteria using three classifiers, which are Support Vector Machine (SVM), 3-Nearest Neighbor (3NN), and Multi-Label *k*-Nearest Neighbor (ML-*k*NN) [46,47]. The 13 multi-label benchmark data sets utilized in the experiments are depicted first. Following that, the findings of the experiments are discussed and examined. Four evaluation metrics that we employed in the experiments have been offered in Section 2.2. The approximate experimental framework is depicted in Figure 4.

### 5.1. Multi-Label Data Sets

A total of 13 multi-label benchmark data sets from 4 different domains have been depicted in Table 3, which are collected on the Mulan repository [48]. Among them, the Birds data set classifies the birds in Audio [49], the Emotions data set is gathered for Music [38], the Genbase and Yeast data sets are primarily concerned with the Biology category [34], and the remaining 9 data sets are categorized as Text. The 13 data sets we chose have an abundant number of instances, which are split into two parts: training set and test set [48]. Ueda and Saito [50] attempted to classify real Web pages linked from the “yahoo.com” domain, which is composed of 14 top-level categories, each of which is split into many second-level subcategories. They tested 11 of the 14 independent text classification problems by focusing on the second-level categories. For each problem, the training set includes 2000 documents and the test set includes 3000 documents, such as the Arts and Health data sets, and so on [51]. The number of labels and the number of features both vary substantially. Previous research demonstrates that maintaining 10% of the features results in no loss, while retaining 1% of the features results in a slight loss dependent on document frequency [3]. For example, the Arts and Social data sets have more than 20,000 features and 50,000 features, respectively, and they retain about 2% of the features with the highest document frequency. The continuous features of 13 data sets are discretized into equal intervals with 3 bins as indicated in the literature [38,52].

### 5.2. The Theoretical Justification of TCRFS on an Artificial Data Set

To further justify the indispensability of the three key aspects (candidate features, selected features, and label correlations) for feature relevance evaluation. We employ an artificial data set to compare the classification performance of five information-theoretical-based methods (D2F, PMU, SCLS, MUCO, and TCRFS) that use distinct feature relevance terms. With respect to the feature relevance terms, D2F and PMU employ the amount of information between candidate features and labels, SCLS employs a scalable relevance evaluation, which takes feature redundancy into account in feature relevance, MUCO employs fuzzy mutual information, and TCRFS comprehensively considers the three types of conditional relevance we mentioned to design FR. Table 4 and Table 5 display the training set and the test set, respectively.

Table 6 shows the experimental results and the feature ranking of each approach on the artificial data set. As shown in Table 6, the first feature selected by TCRFS is $f_{5}$. Different from D2F and PMU, $f_{2}$ is regarded as the least essential feature. In TCRFS, feature rankings $f_{0},f_{8}$, and $f_{4}$ are higher than the feature ranking of SCLS, whereas MUCO selects $f_{4}$ as the first feature. TCRFS achieves the best classification performance overall. Therefore, TCRFS, which considers three key aspects (candidate features, selected features, and label correlations), is justified.

### 5.3. Analysis and Discussion of the Experimental Findings

The experiments that run on a 3.70 GHz Intel Core i9-10900K processor with 32 GB of main memory are performed on four different evaluation criteria regarding three classifiers. Python is used to create the proposed method [53]. Hamming Loss is conducted on the ML-*k*NN (*k* = 10) classifier, and Macro-$F_{1}$ and Micro-$F_{1}$ measures are conducted on the SVM and 3NN classifiers. The number of selected features on the 12 data sets is set to {1%, 2%,..., 20%} of the total number of features when using a step size of 1, whereas the number of selected features on the Medical data set is set to {1%, 2%,..., 17%}. Table 7, Table 8, Table 9, Table 10, Table 11 and Table 12 present the classification performance of 6 contrasted approaches and TCRFS on 13 data sets. The average classification results and standard deviations are used to express the classification performance. The average classification results of each method on all data sets are represented in the row “Average”. The data of the best-performing classification results in Table 7, Table 8, Table 9, Table 10, Table 11 and Table 12 are bolded.

**Figure 5:**
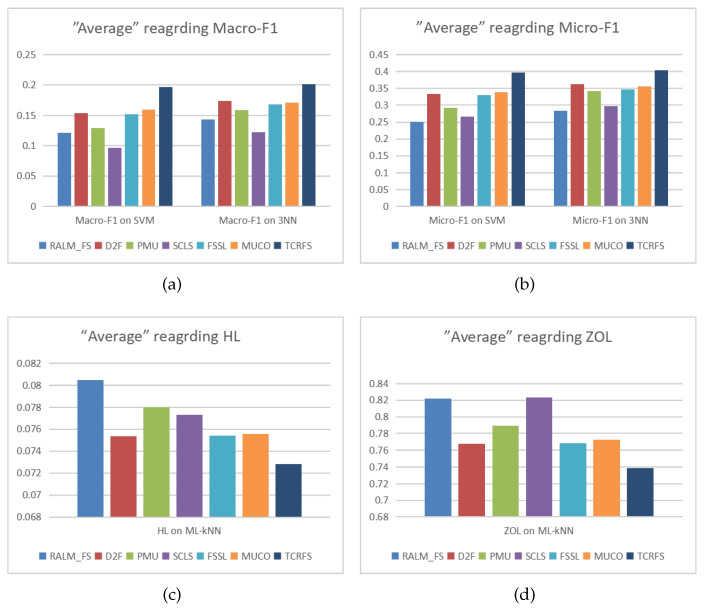
The average values of each method for (**a**) Macro-$F_{1}$, (**b**) Micro-$F_{1}$, (**c**) HL, (**d**) ZOL.

Observing Table 7 and Table 8, TCRFS delivers the optimum classification performance on SVM classifier regarding Macro-$F_{1}$ and Micro-$F_{1}$ measures, since the higher the values of the two measures, the more superior the classification performance. In Table 9, except for the Yeast data set, TCRFS beats 6 other contrasted approaches on 12 data sets using 3NN classifier for Macro-$F_{1}$. TCRFS surpasses the other 6 contrasted approaches on 11 data sets using the 3NN classifier for Micro-$F_{1}$ in Table 10. According to the properties of the HL and ZOL measures, the lower values of the two measures mean the more excellent classification performance. In Table 11 and Table 12, TCRFS can exhibit the best system performance on 11 data sets on the ML-*k*NN classifier for the HL and ZOL criteria. In some cases, comprehensive consideration of the three key aspects to assess feature relevance does not achieve the best classification effect. The classification results of D2F takes the first position on the Yeast data set regarding Macro-$F_{1}$ on the 3NN classifier. PMU and RALM-FS possess the optimal classification performance on the Yeast data set and the Education data sets, respectively. In terms of HL (Table 11), RALM-FS and SCLS surpass other approaches on the Birds and Emotions data sets, respectively. In terms of ZOL (Table 12), FSSL and D2F surpass other approaches on the Birds and Emotions data sets, respectively. Despite the fact that D2F, PMU, RALM-FS, SCLS and FSSL have the greatest system performance on individual data sets, the overall optimal classification performance is still TCRFS. The average values of each method for different evaluation criteria are illustrated in Figure 5. The abscissa and different colored bars represent different feature selection methods, while the ordinate represents the average value.

**Figure 6:**
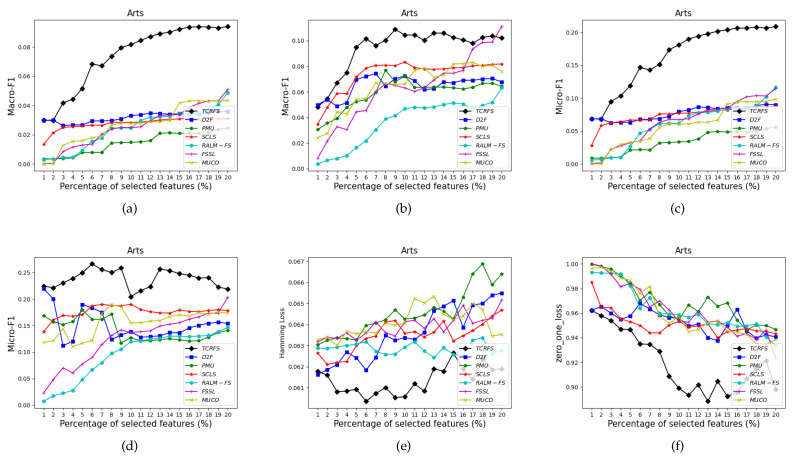
The classification performance of seven methods on Arts data set for (**a**) Macro-$F_{1}$ using SVM, (**b**) Macro-$F_{1}$ using 3NN, (**c**) Micro-$F_{1}$ using SVM, (**d**) Micro-$F_{1}$ using 3NN, (**e**) HL using ML-*k*NN, (**f**) ZOL using ML-*k*NN.

**Figure 7:**
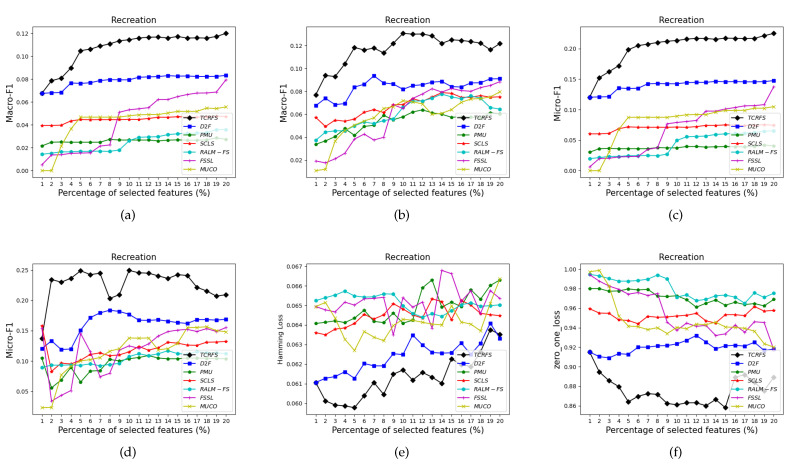
The classification performance of seven methods on Recreation data set for (**a**) Macro-$F_{1}$ using SVM, (**b**) Macro-$F_{1}$ using 3NN, (**c**) Micro-$F_{1}$ using SVM, (**d**) Micro-$F_{1}$ using 3NN, (**e**) HL using ML-*k*NN, (**f**) ZOL using ML-*k*NN.

**Figure 8:**
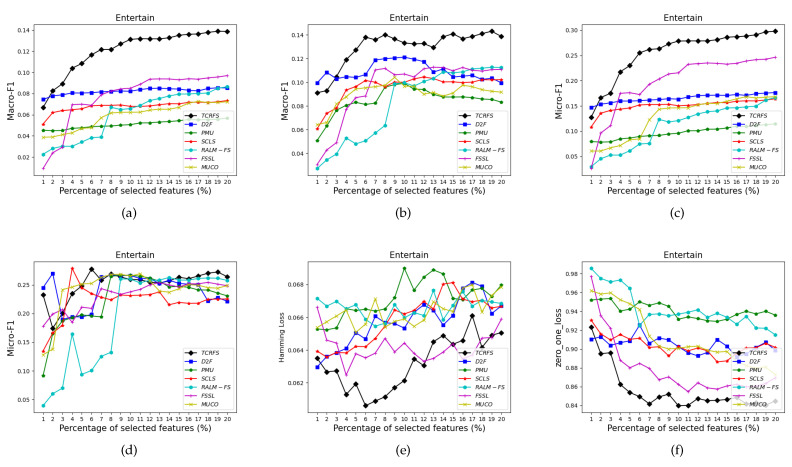
The classification performance of seven methods on Entertain data set for (**a**) Macro-$F_{1}$ using SVM, (**b**) Macro-$F_{1}$ using 3NN, (**c**) Micro-$F_{1}$ using SVM, (**d**) Micro-$F_{1}$ using 3NN, (**e**) HL using ML-*k*NN, (**f**) ZOL using ML-*k*NN.

**Figure 9:**
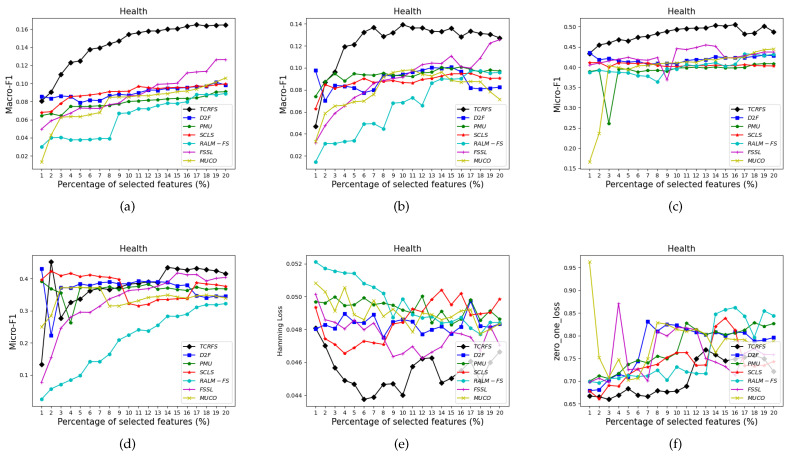
The classification performance of seven methods on Health data set for (**a**) Macro-$F_{1}$ using SVM, (**b**) Macro-$F_{1}$ using 3NN, (**c**) Micro-$F_{1}$ using SVM, (**d**) Micro-$F_{1}$ using 3NN, (**e**) HL using ML-*k*NN, (**f**) ZOL using ML-*k*NN.

Observing the trend of the bar graphs in Figure 5a,b, Macro-$F_{1}$ results and Micro-$F_{1}$ results achieved on the SVM classifier and 3NN classifier have reached similar classification performance. The average results of TCRFS in terms of Macro-$F_{1}$ are roughly 0.2 or above, and the average results of TCRFS in terms of Micro-$F_{1}$ are roughly 0.4 or above, which are clearly greater than the average results of other approaches. The average result of TCRFS is less than 0.074 in Figure 5c and less than 0.74 in Figure 5d, which are clearly less than the average results of other approaches. Intuitively, TCRFS clearly presents the most excellent average values in terms of the four evaluation criteria. In order to further observe the classification performance of the seven methods on the data sets, we draw Figure 6, Figure 7, Figure 8 and Figure 9.

Figure 6, Figure 7, Figure 8 and Figure 9 indicate that TCRFS delivers superior classification performance on the Arts, Recreation, Entertain, and Health data sets regarding the four evaluation criteria. As shown in Figure 6, the classification performance of our method is significantly better than the other six contrasted methods. On the Recreation data set (Figure 7), the classification performance of the method is not constantly improved by increasing the number of selected features. TCRFS, for example, may obtain the most significant classification results regarding the ZOL measure when the number of selected features is set at 8% or 11% of the total number of features. On the Entertain data set (Figure 8), TCRFS is clearly in the lead regarding Macro-$F_{1}$ when the percentage of the selected features is larger than one. In terms of HL and ZOL, TCRFS also possesses significant advantages among the seven methods. The proposed method can obtain the optimum classification performance for each metric when the percentage of the selected features is set to 6%. In Figure 9, our method outperforms the other six contrasted methods on the Health data set utilizing the four metrics. Although in most cases the performance of feature selection methods improves as the number of selected features increases, as the number of features increases to a certain number, the improvement in the classification performance tends to be flat. When the percentage of the number of features increases to about 16% on the Arts data set (Figure 6a–d) and the percentage of the number of features increases to about 19% on the Entertain data (Figure 8a–d), the classification performance has reached a relatively high level. That is to say, an optimal feature subset is to select a smaller number of features to achieve a better classification performance. However, some methods appear to have the same classification performance as TCRFS in Figure 8d and Figure 9e, but TCRFS is superior on average, and they are not as excellent as TCRFS overall. As a consequence, it is critical to consider the three types of conditional relevance for multi-label feature selection.

We create the final feature subset by starting from an empty feature subset and adding a feature after each calculation of the proposed method. According to the TCRFS evaluation function, the score of each candidate feature is calculated and sorted. Due to TCRFS using three incremental information terms as the evaluation criteria for feature relevance, the incremental information of the remaining candidate features will change after each time the selection operation of candidate features is completed. It needs to be recalculated and scored. Therefore, while achieving better classification performance, more time is consumed.

## 6. Conclusions

In this paper, a TCRFS that combines FR and LR is proposed to capture the optimal selected feature subset. FR fuses three incremental information terms that take three key aspects into consideration to convey three types of conditional relevance. Then, TCRFS is compared with 1 embedded approach (RALM-FS) and 5 information-theoretical-based approaches (D2F, PMU, SCLS, FSSL, and MUCO) on 13 multi-label benchmark data sets to demonstrate its efficacy. The classification performance of seven multi-label feature selection methods is evaluated through four multi-label metrics (Macro-$F_{1}$, Micro-$F_{1}$, Hamming Loss, and Zero One Loss) for three classifiers (SVM, 3NN, and ML-*k*NN). Finally, the classification results verify that TCRFS outperforms the other six contrasted approaches. Therefore, candidate features, selected features, and label correlations are critical for feature relevance evaluation, and they can aid in the selection of a more suitable subset of selected features. Our current research is based on a fixed label set for multi-label feature selection. In our future research, we intend to explore multi-label feature selection integrating information theory with the stream label problem.

## Figures and Tables

**Table 1:** Abbreviations meaning statistics.

Abbreviations	Corresponding Meanings
FR	A novel feature relevance term
LR	A label-related feature redundancy term
TCRFS	Feature Selection combining three types of Conditional Relevance

**Table 2:** Feature relevance terms and feature redundancy terms of multi-label feature selection methods.

Methods	Feature Relevance Terms	Feature Redundancy Terms
D2F	$\sum_{l_{i}\in L}\;I\left(f_{k};l_{i}\right)$	$\sum_{f_{j}\in S}\sum_{l_{i}\in L}I\left(f_{k};f_{j};l_{i}\right)$
PMU	$\sum_{l_{i}\in L}\;I\left(f_{k};l_{i}\right)$	$\sum_{f_{j}\in S}\sum_{l_{i}\in L}I\left(f_{k};f_{j};l_{i}\right)+\sum_{l_{i}\in L}\sum_{l_{j}\in L}I\left(f_{k};l_{i};l_{j}\right)$
SCLS	$\left[1-\sum_{f_{j}\in S}\frac{I(f_{k};f_{j})}{H(f_{k})}\right]\sum_{l_{i}\in L}I\left(f_{k};l_{i}\right)$	None
MUCO	$FMI(f_{k};L)$	$\frac{1}{\left|S\right|}\sum_{f_{j}\in S}\left(FMI\left(f_{k};f_{j}\right)\right)$
TCRFS	$\frac{1}{\left|L||S\right|}\sum_{l_{i}\in L}\sum_{f_{j}\in S}\left[I\left(f_{k};l_{i}|f_{j}\right)+I\left(f_{j};l_{i}|f_{k}\right)\right]+\frac{1}{\left|L||L-1\right|}\sum_{l_{i}\in L}\sum_{i\neq j,l_{j}\in L}I\left(f_{k};l_{i}|l_{j}\right)$	$\frac{1}{\left|L||L-1\right|}\sum_{l_{i}\in L}\sum_{f_{j}\in S}\left[I\left(f_{k};f_{j}\right)-I\left(f_{k};f_{j}|l_{i}\right)\right]$

**Table 3:** The depiction of data sets in our experiments.

No.	Data Set	#Domains	#Labels	#Features	#Training	#Test	#Instance
1	Birds	Audio	19	260	322	323	645
2	Emotions	Music	6	72	391	202	593
3	Genbase	Biology	27	1185	463	199	662
4	Yeast	Biology	14	103	1500	917	2417
5	Medical	Text	45	1449	333	645	978
6	Entertain	Text	21	640	2000	3000	5000
7	Recreation	Text	22	606	2000	3000	5000
8	Arts	Text	26	462	2000	3000	5000
9	Health	Text	32	612	2000	3000	5000
10	Education	Text	33	550	2000	3000	5000
11	Reference	Text	33	793	2000	3000	5000
12	Social	Text	39	1047	2000	3000	5000
13	Science	Text	40	743	2000	3000	5000

**Table 4:** Training set.

$f_{0}$	$f_{1}$	$f_{2}$	$f_{3}$	$f_{4}$	$f_{5}$	$f_{6}$	$f_{7}$	$f_{8}$	$f_{9}$	$y_{0}$	$y_{1}$	$y_{2}$	$y_{3}$
1	1	0	0	0	0	1	0	1	0	1	0	0	1
0	0	0	1	1	0	1	0	0	0	1	1	1	1
0	1	0	1	0	0	0	0	1	0	0	0	1	1
0	1	0	0	1	0	0	1	0	1	1	0	0	0
1	1	1	0	0	1	0	1	1	0	0	0	0	0
1	0	0	0	0	0	1	0	1	0	1	1	0	0
1	0	0	0	1	0	1	0	1	0	0	1	0	1
0	0	1	0	1	0	0	1	0	1	0	0	0	0
0	1	0	1	0	1	0	0	0	0	0	1	1	0
0	1	1	0	0	0	0	0	1	0	1	0	0	1
1	1	0	0	0	0	1	1	1	0	1	1	0	1
1	1	0	1	1	0	0	1	0	0	1	0	0	0
0	1	1	1	0	0	0	0	0	0	0	1	1	0
0	1	1	0	1	0	0	1	0	1	1	0	0	0
1	1	0	0	0	1	0	1	1	0	0	1	1	0
1	0	1	0	0	0	0	0	1	0	1	1	0	1
0	0	0	0	1	0	1	0	1	0	0	0	1	0
0	0	1	0	1	0	0	1	0	1	0	0	0	1
0	0	0	1	0	1	0	0	0	0	0	1	0	0
0	1	1	0	1	0	0	1	1	1	1	1	0	0

**Table 5:** Test set.

$f_{1}$	$f_{2}$	$f_{3}$	$f_{4}$	$f_{5}$	$f_{6}$	$f_{7}$	$f_{8}$	$f_{9}$	$f_{10}$	$y_{1}$	$y_{2}$	$y_{3}$	$y_{4}$
1	1	0	0	0	0	1	0	1	1	0	1	1	0
0	0	1	1	1	0	1	0	1	1	0	0	1	0
1	0	1	1	0	1	0	1	0	0	0	1	0	1
1	1	0	0	0	1	0	0	0	0	0	0	1	0
1	0	0	1	1	0	0	1	0	1	0	1	1	0
1	0	1	0	1	1	0	0	1	1	0	1	0	1
1	1	0	0	0	0	1	0	1	0	0	1	1	1
0	0	1	0	1	0	1	1	1	1	1	0	1	0
1	0	1	1	1	1	0	0	0	0	0	1	0	0
0	1	0	0	0	1	0	0	0	0	1	1	0	1

**Table 6:** Experimental results on the artificial data set.

Methods	Feature Ranking	SVM		ML-*k*NN			
		Macro-$F_{1}$ ↑	Micro-$F_{1}$ ↑	Macro-$F_{1}$ ↑	Micro-$F_{1}$ ↑	HL ↓	ZOL ↓
TCRFS	$\left\{f_{5},f_{0},f_{7},f_{8},f_{3},f_{4},f_{1},f_{6},f_{9},f_{2}\right\}$	**0.332**	**0.457**	**0.375**	**0.435**	**0.5000**	**0.97**
D2F	$\left\{f_{5},f_{0},f_{7},f_{8},f_{3},f_{4},f_{1},f_{6},f_{2},f_{9}\right\}$	0.331	0.455	0.374	0.431	0.5150	0.97
PMU	$\left\{f_{5},f_{0},f_{7},f_{8},f_{3},f_{4},f_{1},f_{6},f_{2},f_{9}\right\}$	0.331	0.455	0.374	0.431	0.5150	0.97
SCLS	$\left\{f_{5},f_{9},f_{3},f_{7},f_{0},f_{6},f_{1},f_{2},f_{8},f_{4}\right\}$	0.32	0.409	0.373	0.427	0.5025	0.98
MUCO	$\left\{f_{4},f_{6},f_{7},f_{8},f_{1},f_{2},f_{3},f_{0},f_{5},f_{9}\right\}$	0.331	0.397	0.334	0.385	0.5450	0.98

**Table 7:** Classification performance of each method regarding Macro-$F_{1}$ on SVM classifier (mean ± std).

Data Set	RALM-FS	D2F	PMU	SCLS	FSSL	MUCO	TCRFS
Birds	0.058±0.024	0.077±0.04	0.075±0.036	0.039±0.026	0.049±0.027	0.1±0.051	**0.116±0.058**
Emotions	0.147±0.101	0.315±0.061	0.239±0.095	0.336±0.055	0.35±0.085	0.366±0.127	**0.381±0.089**
Genbase	0.738±0.153	0.706±0.107	0.628±0.093	0.241±0.022	0.762±0.133	0.758±0.14	**0.765±0.129**
Yeast	0.229±0.036	0.258±0.034	0.262±0.031	0.207±0.014	0.213±0.037	0.227±0.044	**0.276±0.036**
Medical	0.129±0.063	0.191±0.055	0.188±0.057	0.079±0.013	0.227±0.086	0.254±0.074	**0.311±0.075**
Entertain	0.059±0.022	0.081±0.006	0.051±0.004	0.067±0.006	0.075±0.028	0.058±0.013	**0.119±0.023**
Recreation	0.024±0.008	0.077±0.009	0.026±0.002	0.044±0.004	0.042±0.024	0.041±0.018	**0.105±0.019**
Arts	0.024±0.014	0.031±0.005	0.014±0.007	0.027±0.005	0.025±0.014	0.026±0.014	**0.072±0.024**
Health	0.062±0.021	0.089±0.008	0.078±0.008	0.089±0.01	0.087±0.022	0.077±0.021	**0.141±0.028**
Education	0.024±0.009	0.046±0.009	0.027±0.008	0.038±0.006	0.041±0.015	0.041±0.019	**0.065±0.013**
Reference	0.023±0.01	0.039±0.004	0.026±0.006	0.024±0.004	0.03±0.011	0.04±0.017	**0.065±0.013**
Social	0.046±0.018	0.07±0.01	0.052±0.012	0.052±0.006	0.055±0.02	0.059±0.019	**0.101±0.028**
Science	0.008±0.006	0.021±0.003	0.009±0.005	0.016±0.004	0.023±0.013	0.024±0.013	**0.049±0.017**
Average	0.121	0.154	0.129	0.097	0.152	0.159	**0.197**

**Table 8:** Classification performance of each method regarding Micro-$F_{1}$ on SVM classifier (mean ± std).

Data Set	RALM-FS	D2F	PMU	SCLS	FSSL	MUCO	TCRFS
Birds	0.096±0.046	0.135±0.075	0.129±0.055	0.06±0.04	0.084±0.049	0.197±0.078	**0.207±0.086**
Emotions	0.178±0.113	0.372±0.038	0.295±0.099	0.422±0.038	0.434±0.06	0.425±0.118	**0.45±0.07**
Genbase	0.958±0.136	0.968±0.066	0.946±0.066	0.541±0.014	0.969±0.108	0.977±0.071	**0.979±0.067**
Yeast	0.552±0.027	0.565±0.023	0.571±0.021	0.532±0.008	0.54±0.026	0.549±0.031	**0.584±0.027**
Medical	0.363±0.147	0.629±0.07	0.625±0.075	0.37±0.009	0.661±0.168	0.711±0.087	**0.753±0.058**
Entertain	0.108±0.043	0.163±0.015	0.096±0.013	0.149±0.016	0.192±0.062	0.127±0.041	**0.251±0.054**
Recreation	0.043±0.018	0.138±0.016	0.038±0.003	0.07±0.007	0.065±0.038	0.077±0.034	**0.198±0.035**
Arts	0.059±0.033	0.075±0.013	0.033±0.016	0.072±0.015	0.062±0.033	0.056±0.031	**0.16±0.051**
Health	0.401±0.018	0.418±0.012	0.391±0.029	0.406±0.004	0.426±0.02	0.396±0.061	**0.479±0.026**
Education	0.073±0.024	0.117±0.017	0.077±0.014	0.138±0.023	0.142±0.056	0.132±0.06	**0.203±0.045**
Reference	0.153±0.077	0.305±0.039	0.265±0.05	0.259±0.039	0.286±0.062	0.314±0.093	**0.344±0.058**
Social	0.252±0.107	0.396±0.072	0.31±0.07	0.384±0.049	0.357±0.105	0.356±0.082	**0.426±0.073**
Science	0.029±0.015	0.053±0.01	0.024±0.016	0.058±0.014	0.071±0.034	0.074±0.037	**0.122±0.032**
Average	0.251	0.333	0.292	0.266	0.33	0.338	**0.397**

**Table 9:** Classification performance of each method regarding Macro-$F_{1}$ on 3NN classifier (mean ± std).

Data Set	RALM-FS	D2F	PMU	SCLS	FSSL	MUCO	TCRFS
Birds	0.093±0.036	0.15±0.066	0.122±0.036	0.078±0.028	0.075±0.037	0.131±0.038	**0.17±0.048**
Emotions	0.312±0.074	0.434±0.033	0.413±0.046	0.426±0.042	0.442±0.124	0.434±0.101	**0.468±0.068**
Genbase	0.689±0.132	0.65±0.086	0.604±0.089	0.224±0.018	0.702±0.12	0.7±0.123	**0.71±0.103**
Yeast	0.3±0.027	**0.348±0.038**	0.34±0.03	0.301±0.026	0.309±0.041	0.314±0.033	0.334±0.039
Medical	0.069±0.029	0.121±0.019	0.114±0.018	0.063±0.006	0.149±0.04	0.155±0.03	**0.184±0.025**
Entertain	0.079±0.031	0.108±0.011	0.083±0.014	0.095±0.013	0.094±0.028	0.089±0.014	**0.128±0.019**
Recreation	0.06±0.014	0.082±0.011	0.053±0.01	0.066±0.011	0.057±0.026	0.057±0.021	**0.114±0.019**
Arts	0.036±0.018	0.064±0.01	0.058±0.014	0.072±0.016	0.061±0.026	0.064±0.019	**0.092±0.02**
Health	0.064±0.027	0.087±0.011	0.093±0.008	0.087±0.011	0.087±0.024	0.08±0.018	**0.122±0.022**
Education	0.047±0.011	0.065±0.009	0.057±0.009	0.059±0.01	0.063±0.015	0.06±0.019	**0.074±0.012**
Reference	0.032±0.01	0.044±0.004	0.034±0.007	0.036±0.005	0.041±0.01	0.046±0.015	**0.07±0.011**
Social	0.052±0.013	0.064±0.006	0.054±0.006	0.051±0.004	0.064±0.024	0.058±0.016	**0.091±0.011**
Science	0.024±0.008	0.04±0.005	0.028±0.008	0.03±0.004	0.039±0.019	0.036±0.011	**0.057±0.012**
Average	0.143	0.174	0.158	0.122	0.168	0.171	**0.201**

**Table 10:** Classification performance of each method regarding Micro-$F_{1}$ on 3NN classifier (mean ± std).

Data Set	RALM-FS	D2F	PMU	SCLS	FSSL	MUCO	TCRFS
Birds	0.171±0.066	0.231±0.072	0.203±0.05	0.144±0.043	0.159±0.054	0.227±0.057	**0.273±0.061**
Emotions	0.353±0.051	0.469±0.02	0.445±0.022	0.46±0.028	0.478±0.114	0.471±0.079	**0.503±0.05**
Genbase	0.956±0.134	0.95±0.061	0.919±0.064	0.518±0.012	0.959±0.126	0.974±0.074	**0.977±0.065**
Yeast	0.529±0.019	0.549±0.041	**0.553±0.014**	0.518±0.035	0.526±0.049	0.523±0.041	0.552±0.041
Medical	0.294±0.108	0.53±0.038	0.522±0.037	0.353±0.013	0.558±0.121	0.591±0.053	**0.638±0.032**
Entertain	0.187±0.085	0.241±0.032	0.22±0.053	0.217±0.031	0.229±0.037	0.234±0.048	**0.249±0.032**
Recreation	0.102±0.014	0.159±0.024	0.094±0.02	0.115±0.017	0.111±0.045	0.112±0.041	**0.224±0.033**
Arts	0.095±0.045	0.15±0.031	0.137±0.028	0.172±0.028	0.126±0.044	0.155±0.029	**0.237±0.028**
Health	0.2±0.097	0.367±0.05	0.361±0.038	0.366±0.064	0.33±0.092	0.339±0.038	**0.38±0.063**
Education	**0.254±0.026**	0.19±0.032	0.18±0.04	0.19±0.033	0.238±0.032	0.191±0.054	0.22±0.036
Reference	0.164±0.073	0.364±0.048	0.35±0.043	0.294±0.048	0.334±0.049	0.319±0.085	**0.42±0.046**
Social	0.302±0.04	0.39±0.051	0.363±0.051	0.368±0.04	0.354±0.069	0.349±0.056	**0.432±0.045**
Science	0.08±0.037	0.123±0.019	0.099±0.018	0.147±0.034	0.112±0.041	0.136±0.037	**0.153±0.031**
Average	0.284	0.363	0.342	0.297	0.347	0.355	**0.404**

**Table 11:** Classification performance of each method regarding HL on ML-*k*NN classifier (mean ± std).

Data Set	RALM-FS	D2F	PMU	SCLS	FSSL	MUCO	TCRFS
Birds	**0.05081±0.00106**	0.05269±0.00164	0.05227±0.0017	0.0544±0.00188	0.0526±0.00143	0.05138±0.00133	0.05147±0.00103
Emotions	0.33752±0.01318	0.29408±0.01324	0.31854±0.00914	**0.27947±0.00716**	0.2922±0.01356	0.28878±0.02079	0.28012±0.01018
Genbase	0.00377±0.0068	0.00315±0.00391	0.00469±0.00405	0.03093±0.00042	0.00301±0.00585	0.00296±0.00433	**0.00269±0.00396**
Yeast	0.23706±0.00434	0.22784±0.00287	0.22793±0.00356	0.2332±0.00431	0.23182±0.00293	0.23341±0.00377	**0.22565±0.00404**
Medical	0.02702±0.0007	0.01955±0.00105	0.01972±0.00107	0.02332±0.00018	0.01842±0.00237	0.01852±0.00108	**0.01774±0.0009**
Entertain	0.06652±0.00057	0.06568±0.00133	0.06708±0.00112	0.06587±0.00144	0.06415±0.00103	0.06631±0.00085	**0.06315±0.00145**
Recreation	0.06513±0.00038	0.06239±0.00077	0.06484±0.00068	0.06444±0.0006	0.06513±0.00069	0.06419±0.0007	**0.06144±0.00111**
Arts	0.06285±0.00023	0.0635±0.00122	0.06441±0.00104	0.06339±0.00074	0.06389±0.00057	0.06412±0.00075	**0.06135±0.00063**
Health	0.04969±0.00132	0.04831±0.00051	0.04934±0.00059	0.04848±0.00114	0.04764±0.00101	0.04898±0.00068	**0.04545±0.00111**
Education	0.04414±0.00034	0.04427±0.00073	0.04453±0.00082	0.04408±0.00101	0.04403±0.0006	0.0444±0.00054	**0.04303±0.00069**
Reference	0.03503±0.00035	0.03223±0.00117	0.03357±0.00095	0.0329±0.00021	0.03262±0.00068	0.03332±0.00061	**0.03133±0.00075**
Social	0.03061±0.00122	0.03032±0.00046	0.03091±0.00031	0.02866±0.0007	0.02906±0.00092	0.02967±0.00055	**0.02766±0.00077**
Science	0.03615±0.00028	0.03579±0.0004	0.03626±0.00036	0.03583±0.00041	0.03567±0.00027	0.0361±0.00058	**0.03543±0.00042**
Average	0.08048	0.07537	0.07801	0.07731	0.0754	0.07555	**0.07281**

**Table 12:** Classification performance of each method regarding ZOL on ML-*k*NN classifier (mean ± std).

Data Set	RALM-FS	D2F	PMU	SCLS	FSSL	MUCO	TCRFS
Birds	0.53239±0.00619	0.53352±0.01484	0.55013±0.02117	0.53543±0.00551	**0.52745±0.00789**	0.53007±0.00864	0.54019±0.01396
Emotions	0.92468±0.03724	**0.82815±0.02803**	0.88331±0.05054	0.85502±0.03048	0.85431±0.03856	0.83982±0.03592	0.83522±0.02541
Genbase	0.07909±0.15179	0.06976±0.07896	0.09236±0.07004	0.56379±0.01154	0.06285±0.12667	0.06058±0.07839	**0.05795±0.0815**
Yeast	0.94729±0.02727	0.88602±0.02723	0.89168±0.02807	0.91671±0.01147	0.9233±0.03139	0.91613±0.03483	**0.88586±0.01848**
Medical	0.86604±0.07297	0.65611±0.03702	0.66257±0.04058	0.82617±0.00642	0.62048±0.0981	0.61537±0.0484	**0.58932±0.0373**
Entertain	0.94447±0.01955	0.90565±0.01002	0.94136±0.00863	0.90345±0.01303	0.88309±0.03407	0.91441±0.02957	**0.85752±0.02652**
Recreation	0.97955±0.01057	0.92066±0.00898	0.97122±0.00609	0.95327±0.00543	0.95681±0.02178	0.9493±0.02212	**0.87796±0.01967**
Arts	0.96399±0.0181	0.9548±0.01101	0.97061±0.0167	0.9529±0.01086	0.96364±0.02175	0.96234±0.02165	**0.92196±0.02549**
Health	0.7561±0.0662	0.77159±0.05271	0.77152±0.04486	0.73661±0.0437	0.74891±0.05006	0.7876±0.05694	**0.70867±0.04394**
Education	0.95281±0.0162	0.94833±0.00936	0.95489±0.01428	0.9339±0.01388	0.94176±0.02666	0.93868±0.02975	**0.90171±0.02493**
Reference	0.90776±0.05755	0.80313±0.03802	0.81068±0.05208	0.8284±0.0372	0.80829±0.04754	0.80433±0.0658	**0.7591±0.06182**
Social	0.84735±0.07255	0.73236±0.08727	0.77499±0.06847	0.74463±0.04251	0.75138±0.08065	0.76243±0.052	**0.72314±0.05028**
Science	0.98663±0.00642	0.9725±0.00583	0.98477±0.00815	0.95488±0.01192	0.95139±0.01995	0.96111±0.02084	**0.94441±0.0112**
Average	0.82217	0.76789	0.78924	0.82347	0.76874	0.77247	**0.73869**

## Data Availability

The multi-label data sets used in the experiment are from Mulan Library http://mulan.sourceforge.net/datasets-mlc.html, accessed on 24 November 2021.
